# RNA Interference Suppression of v-ATPase B and Juvenile Hormone Binding Protein Genes Through Topically Applied dsRNA on Tomato Leaves: Developing Biopesticides to Control the South American Pinworm, *Tuta absoluta* (Lepidoptera: Gelechiidae)

**DOI:** 10.3389/fphys.2021.742871

**Published:** 2021-11-18

**Authors:** Govindaraju Ramkumar, Ramasamy Asokan, N. R. Prasannakumar, B. Kariyanna, Sengodan Karthi, Mona S. Alwahibi, Mohamed Soliman Elshikh, Ahmed Abdel-Megeed, Aml Ghaith, Sengottayan Senthil-Nathan, Kandaswamy Kalaivani, Wayne Brian Hunter, Patcharin Krutmuang

**Affiliations:** ^1^Division of Biotechnology, ICAR-Indian Institute of Horticultural Research (IIHR), Bengaluru, India; ^2^Division of Entomology and Nematology, ICAR-Indian Institute of Horticultural Research (IIHR), Bengaluru, India; ^3^Division of Biopesticides and Environmental Toxicology, Sri Paramakalyani Center for Excellence in Environmental Sciences, Manonmaniam Sundaranar University, Tirunelveli, India; ^4^Department of Botany and Microbiology, College of Science, King Saud University, Riyadh, Saudi Arabia; ^5^Department of Plant Protection, Faculty of Agriculture Saba Basha, Alexandria University, Alexandria, Egypt; ^6^Department of Zoology, Faculty of Science, Derna University, Derna, Libya; ^7^Post Graduate and Research Center, Department of Zoology, Sri Parasakthi College for Women, Tirunelveli, India; ^8^U.S. Horticultural Research Laboratory, United States Department of Agriculture, Agricultural Research Service, Fort Pierce, FL, United States; ^9^Department of Entomology and Plant Pathology, Faculty of Agriculture, Chiang Mai University, Chiang Mai, Thailand; ^10^Innovative Agriculture Research Center, Faculty of Agriculture, Chiang Mai University, Chiang Mai, Thailand

**Keywords:** tomato leaf miner, v-ATPase, juvenile hormone binding protein, JHBP, dsRNA, RNAi, lepidoptera, pest management

## Abstract

The South American pinworm *Tuta absoluta* (Meyrick) (Family: Gelechiidae) is one of the most devastating lepidopteran pests in the developing countries of South America, Africa, and Asia. This pest is classified as the most serious threat for tomato production worldwide. In the present study, we analyzed RNAi-mediated control through exogenously applied dsRNA delivery on tomato. The dsRNA treatments were made to target the juvenile hormone binding protein and the v-ATPase B. Both mRNA targets were cloned, validated by sequencing, and used to produce each dsRNA. After treatments the relative transcript expression was analyzed using qRTPCR to assess to efficacy of RNAi. A leaf-dip assay was used to provide late 2nd instar larvae three feeding access periods: 24, 48, and 72 h, to evaluate the effect of gene silencing of each target. Larvae were fed tomato leaves coated with five different RNAi concentrations (10, 20, 30, 40, and 50 micrograms/centimeter-squared), that suppressed two genes (juvenile hormone protein, JHBP, and vacuolar-type adenosine triphosphatase enzyme, v-ATPase). Treatments with dsRNA showed a significant increase in mortality at 24, 48, and 72 h after ingestion (*P* < 0.01, α = 0.05), along with reduced leaf damage, and increased feeding deterrence. The results suggest that these two RNAi products may provide a suitable treatment for control of this and other lepidopteran pests.

## Introduction

*Tuta absoluta* Meyrick (Lepidoptera: Gelechiidae) is an oligophagous pest infesting many Solanaceous crops (Global Distribution Map: CABI, Campos et al., 2017; Biondi et al., 2018; Rwomushana et al., 2019). Since the 1960s, this moth has become one of the key pests of tomato in South America (Garcia and Espul, 1982). In Europe, *T. absoluta* was first reported in Spain in late 2006. Thereafter, it was reported in many countries including India (Shashank et al., 2014). Cost-benefit analysis showed that *T. absoluta* significantly increased costs of pest management. Its primary host is tomato, although potato, brinjal, common bean, and various wild Solanaceous plants are also suitable hosts. Synthetic pesticides are commonly used for pest control worldwide (Guedes et al., 2019). Application of these chemicals against *T. absoluta* has been reported with little success, mainly because the pest has developed resistance (Siqueira et al., 2000; Senthil-Nathan, 2020). Efforts to develop botanical pesticides, like citrus peel extract have met with limited success (Senthil-Nathan, 2013; Miresmailli and Isman, 2014; Campolo et al., 2017). Development of alternative methods for pest control like RNAi biopesticides provide highly specific pesticides that do not harm parasitoids, pollinators, or predators (Chen et al., 2018; Niu et al., 2018; Christiaens et al., 2020; Fletcher et al., 2020; Kunte et al., 2020; Taning et al., 2020; Yan et al., 2020a,b; Sarmah et al., 2021). Research also shows that RNAi biopesticides can be exogenously applied in liquid sprays (Dalakouras et al., 2016; Koch et al., 2016; McLoughlin et al., 2018; Dubrovina and Kiselev, 2019; Dubrovina et al., 2019, 2020; Jalaluddin et al., 2019; Mezzetti et al., 2020) or bound with a carrier-like clay or nanotubes in sprays (Worrall et al., 2019; Fletcher et al., 2020) or applied in water or clay pellets as a soil treatment (Ghosh et al., 2018) or as plant-expressed silencing, which has met with significant success in field crops (Younis et al., 2014; Bramlett et al., 2020; Das and Sherif, 2020; Veillet et al., 2020).

Juvenile Hormone (JH) is essential for regulating the maturation, reproduction, and development of insects. JH inhibits the metamorphosis, leading to growth arrest of insects in their pre-metamorphosis stage, induces insect diapauses, and affects the migratory behavior of insects. It is transported into the target cells *via* JH binding protein (JHBP) (Gilbert et al., 2000). It is present throughout late embryonic and larval development. Changing ratios of JH/20E regulate molting to the next developmental stage and thereby allow for continued growth of insect larvae (Riddiford, 1994).

Insect vacuolar ATPase synthase genes have been compared across many insect orders (Pan et al., 2017). RNA interference, which prompts specific gene silencing through the delivery of homologous double-stranded RNA (dsRNA) fragments, is referred to as the “trigger” (Fire et al., 1998; Mello and Conte, 2004). Application of RNAi to manage insect pests or viral pathogens is widely supportive of integrated pest management strategies and shows great potential (Bramlett et al., 2020; Christiaens et al., 2020; Das and Sherif, 2020). Sarmah et al. (2021) report that while *Tuta absoluta* is sensitive to RNAi treatments demonstrating significantly increased mortality when ingesting the dsRNA made to the *alpha*COP (αCOP) (Coatomer subunit alpha protein) mRNA transcript, there was no significant increase in mortality when treated individuals were fed on by the mirid predator *Nesidiocoris tenuis* (Hemiptera: Miridae). Thus, they concluded that RNAi-mediated control of *T. absoluta* would be a safe addition to biological control programs as it would not negatively affect the pest's natural enemies (Sarmah et al., 2021).

Functional gene studies have provided significant advances in understanding insect physiology across many orders of arthropods including Coleoptera, Diptera, Hemiptera, Hymenoptera, and Lepidoptera (Chaitanya et al., 2017). In the present study we have chosen to silence the genes of v-ATPase B and JHBP, which are involved in the growth and development of insects, as effective targets for the management of pests.

## Materials and Methods

### Insect Rearing and Maintenance

*Tuta absoluta* was obtained from the Division of Biotechnology, ICAR-Indian Institute of Horticultural Research, Bangalore, Karnataka, India. The cultures were maintained on tomato leaves at a 28 ± 1°C temperature, 60–70% relative humidity, and 14:10 h of light: dark photoperiod in the laboratory.

### Target Gene Selection

Genes encoding v-ATPase B and JHBP genes were chosen based on previous successful reports of RNAi used for insect control (Chaitanya et al., 2017). Since no sequence information was available for *T. absoluta* genes, degenerative primers were designed based on conserved amino acid sequence regions from aligned homologs of *Plutella xylostella* (JN410829), *Manduca sexta* (S56567), and *Bombyx mori* (NM_001043483). Based on these orthologous genes, the mRNA for the complete v-ATPase B coding sequence was estimated to be around 1,500 bp (Supplementary Table 1).

### Target Gene Amplification, Template Cloning, and Sequencing

Target transcripts were amplified from cDNA using a nested PCR-based method with degenerate primer pairs in a 20 μL reaction containing 2 μg of cDNA, 3 mM of MgCl^2^, 100 μM of dNTP, 1 μM of each primer, and 2 U of Taq DNA polymerase (Bioline reagents, Germany). Amplifications were done in a Pepseq^TM^ thermo cycler, programmed to cycle at 95°C for 5 min, and followed by 35 cycles of 95°C for 30 s, 60°C for 30 s, 72°C for 15 s, and a final cycle at 72°C for 10 min. Amplification products were analyzed by gel electrophoresis (1.5% agarose gels, voltage 100 mV, for 30 min); fragments were excised, purified using a NucleoSpin^®^ Gel and PCR Clean-up kit^TM^ (Fermentas, GmbH, Germany), ligated into a TA cloning vector (PTZ57R/TB) (GenJET™ Plasmid MiniPrep kit), and used to transform the DH5α *E. coli* strain as per the manufacturer's protocol. After blue-white colonies were screened, plasmids were isolated from “white” colonies using a GenJET™ Plasmid MiniPrep kit (Fermentas, GmbH, Germany) and were sequenced (XCelris Labs, Ahmadabad, India).

### Phylogenetic Analysis

The phylogenetic tree analysis was performed using MEGAX 11.0 software in the NCBI public database. Significant Lepidoptera species included, but were not limited to: *Spodoptera littoralis; Plutella xylostella, Helicoverpa armigera* (Noctuidae)*; Bombyx mori* (Bombycidae)*; Galleria mellonella* (Pyralidae); *and Amyelois transitella* (Pyralidae).

### dsRNA Synthesis

Unmodified, canonical syntaxin-1A dsRNA was synthesized using the Ambion^®^ MEGAscript^®^ RNAi Kit (Ref. No. AM1626) per manual instructions. dsRNA was uniquely designed with specific primers along with T7-polymerase promoter sequences. The reaction volume was made up to 50 μl, v-ATPase B and JHBP plasmid clones were used for the DNA template with primer annealing at 63°C /40 s. The amplified products were run on 1.3% agarose gel, expected bands were eluted, and then were used as templates (1 μg) for synthesis of dsRNA following the manufacturer's protocol (Thermo Scientific, Germany). Finally, dsRNAs were quantified using Thermo NanoDrop^TM^ (Thermo Scientific) and verified by agarose gel electrophoresis (Rebijith et al., 2015; Chaitanya et al., 2017). The dsRNA for v-ATPase was 192 nt without T7, and the JHBP was 136 nt (Supplementary Table 1).

### Oral Delivery of dsRNA *T. absoluta*

Tomato leaflet feeding bioassays were carried out as described by Rebijith et al. (2015) and Chaitanya et al. (2017) with slight modifications. Briefly, a fresh and young tomato leaf (*Lycopersicon esculentum* Mill) was rinsed in 1% Triton-X, rinsed with double-ionized distilled water, and then dried and placed on moist cotton in a Petri dish. The leaflets were treated with 200 μl of the solution topically applied with the following dsRNA concentrations (10, 20, 30, 40, and 50 μg/cm^2^) and spread across the leaf surface using a fine hairbrush. The treated leaves were allowed to sit until dry, ~15–20 min. Then, five *T. absoluta* larvae (late 2nd instars) were transferred to cages with the dsRNA-coated leaves, with three leaves per treatment. Experiments were replicated three times for a total of 45 insects per treatment. Control received nuclease free water applied in the same manner. Mortality was recorded after 24, 48, and 72 h of feeding access. Extra cohort cages provided a source of live insects for qPCR analyses that were sampled over time from each treatment concentration.

### RNA Extraction and cDNA Synthesis

RNA was extracted from 100 mg of fourth instar *T. absoluta* larvae using an Isolate II RNA mini kit (Bioline reagents, Germany). RNA were quantified using a NanoDrop^TM^ Lite Spectrophotometer (Thermo Scientific, Germany) and further analyzed by electrophoresis in 1.5% denaturing agarose gels. The cDNA was synthesized using a Bioline kit manufacture protocol, taking 2.0 μg of RNA and adding oligo- (dT)_18_ primers. The mixtures were incubated at 65°C for 5 min and immediately cooled in ice. A total of 5 × reverse transcriptase buffer, 2.5 mM of dNTPs, and 10 U/μl of RNase inhibitor were added into the tube. The mixtures were incubated at 42°C for 90 min. Finally, reverse transcriptase was added and incubated at 72°C for 15 min as per the manufacturer's instructions (Bioline, Germany).

### Gene Expression Analysis

The v-ATPase subunit B and JHBP genes were assessed by RT-qPCR (Supplementary Table 1). All expression studies were carried out following MIQE. Live larvae were used for RNA isolation using the MyTaq™ One-Step RT-PCR Kit (Catalog No. BIO-65049, Meridian Bioscience™) as per the manufacturer's protocol. In brief, samples were diluted 1:5 before RT-qPCR. The final reaction volume was adjusted to 20 μl with RNAse free water and SYBR Green (TaKaRa, Japan). The cDNA from all samples was prepared as normalized concentrations of 5 ng/μl in 10 μl, then diluted 1:5 before RT-qPCR reactions as per the instructions. All selected primers used a 10-μM scale with β-actin (KU872540) as the reference gene (Pfaffl, 2001; Pfaffl et al., 2002). The expressed β actin constitutively was used for loading normalization. RT-qPCR was carried out with the following conditions, *viz*., 95°C for 5 min, followed by 40 amplification cycles at 95°C for 30 s, and 60°C for 1 min in a Light Cycler 480 II (Roche Applied Science, Switzerland). Relative expression was calculated by the 2^−ΔΔct^ method (Pfaffl et al., 2002).

### Statistical Analysis

Probit studies were performed using SPSS v 16.0. The qRT-PCR gene expression data were analyzed using GraphPad Prism 5.0 software (www.graphpad.com). Significant differences analyzed by one-way ANOVA, followed by *post-hoc Tukey (P* ≤ *0.05)*. The correlation between mortality and downregulation was analyzed. T-tests for independent samples or Mann–Whitney U-tests, depending on data distribution, were used to test for significant differences in expression levels (ΔΔ^Ct^ values) of the target genes between the experimental and control.

## Results

### Cloning, Sequencing, and Phylogenetic Analysis

v-ATPase subunit B and JHBP gene cDNA sequencing resulted in 851 and 584 bp products, respectively. NCBI-BLASTX analysis indicated a 98% amino acid sequence match with *Plutella xylostella* sequences (Rebijith et al., 2015). Our target gene sequences and alignments were deposited at NCBI (v-ATPase B, Accession number; MN414200; JHBP Accession number; OK066277). The sequences of v-ATPase B and JHBP were clustered with publicly available sequences from Lepidoptera, BLASTn, NCBI, and the nr database. Significant identified Lepidoptera species in the NCBI public database included *Spodoptera littoralis* (Noctuidae)*; Plutella xylostella* (Plutellidae)*, Helicoverpa armigera* (Noctuidae) (Ni et al., 2017)*; Bombyx mori* (Bombycidae); *Galleria mellonella* (Pyralidae); and *Amyelois transitella* (Pyralidae).

### dsRNA Delivery Using a Coated-Leaf Feeding Assay

**Figure 1:**
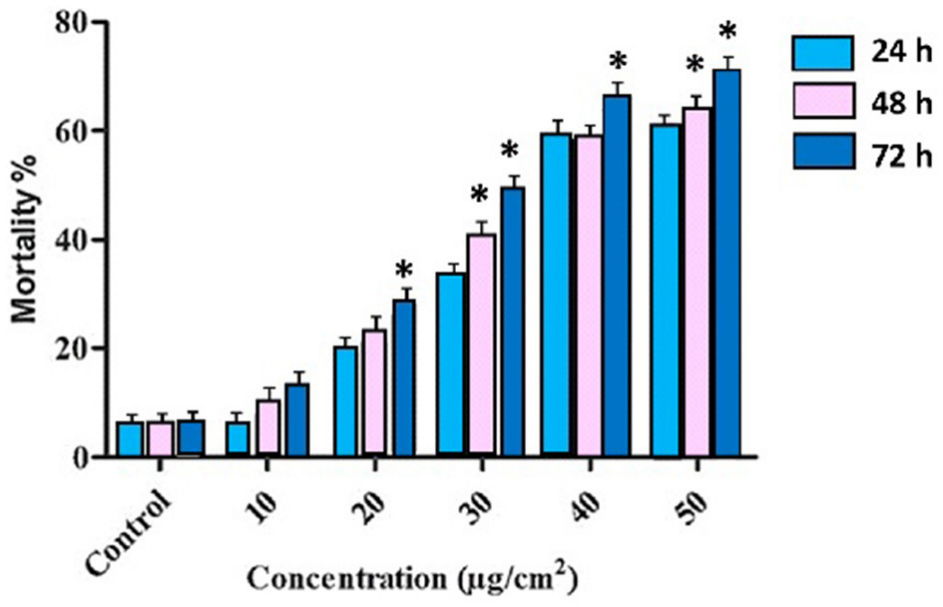
Percentage of mortality rates of *T. absoluta* larvae (late 2nd instars) post ingestion of JHBP dsRNA through treated leaves. Mortality was positively correlated with dsRNA concentration and feeding access time period. The mortality is averaged across three trials each with three biological replicates. The three time-points, 24, 48, and 72 h, were sampled across each dsRNA concentration treatment. One-way ANOVA followed by *post-hoc* Tukey's multiple comparison test were completed. Error bars indicate standard errors of each mean values. Asterisk (*) shows statistically significant at various time intervals at different concentrations (*P* < 0.05).

**Figure 2:**
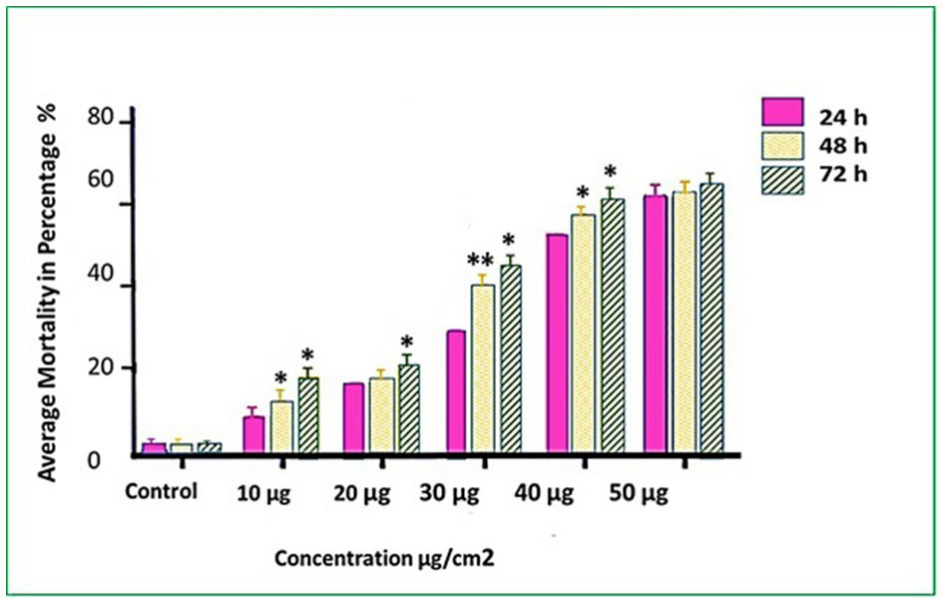
Mortality rates (%) of *T. absoluta* larvae (late 2nd instars) post feeding access period on v-ATPase B dsRNA-treated leaves. Observed mortality was recorded every 24, 48, and 72 h. The average values were obtained over three biological replications. One-way ANOVA followed with Tukey's multiple comparison (*P* < 0.05) were completed. Error bars are the SE ± means-averaged mortality across three trials each with three biological replicates. Asterisk shows statistically significant in various time intervals at different concentrations (^*^P < 0.05 and ^**^P < 0.01).

**Figure 3:**
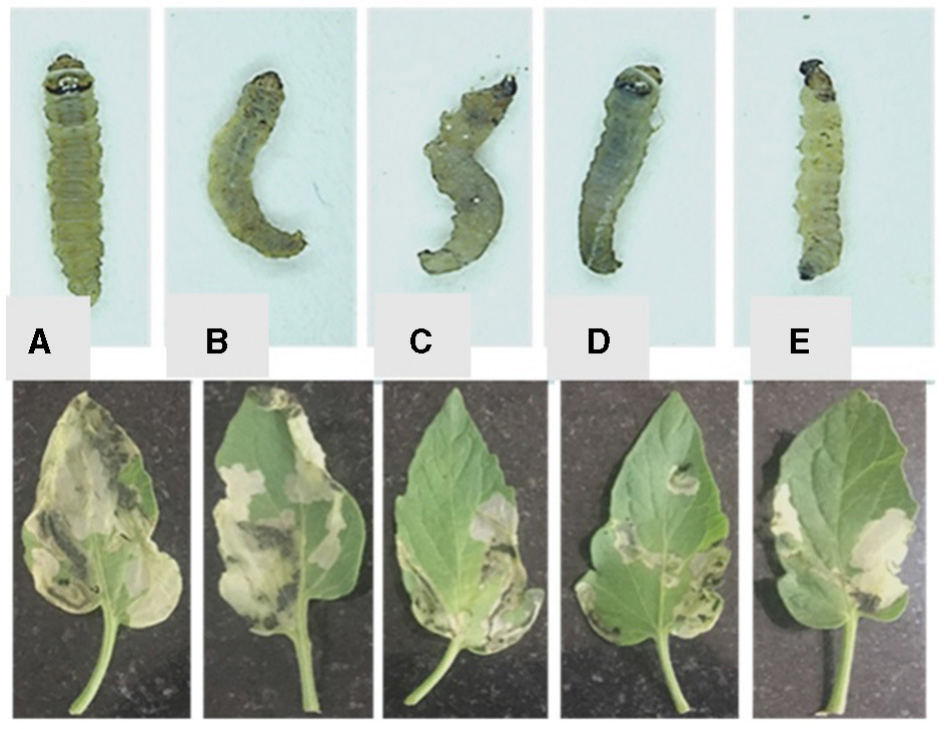
Single leaf feeding assay. There was an observed correlation between decreased feeding damage to leaves with increasing dsRNA concentrations **(A)** 10 μg dsRNA /cm^2^, up to **(E)** 50 μg dsRNA /cm^2^. Individual leaves were coated with dsRNA solution at different concentrations of: **(A)** 10; **(B)** 20; **(C)** 30; **(D)** 40; **(E)** 50 μg/cm^2^ leaf. The dsRNA concentration of treated leaves at 10 μg/cm^2^ had >50% of the leaf surface eaten, and larger larvae than the 50 μg/cm^2^ treatment, while the dsRNA concentrations of 20–50 (μg/cm^2^) had <50% leaf damage.

**Figure 4:**
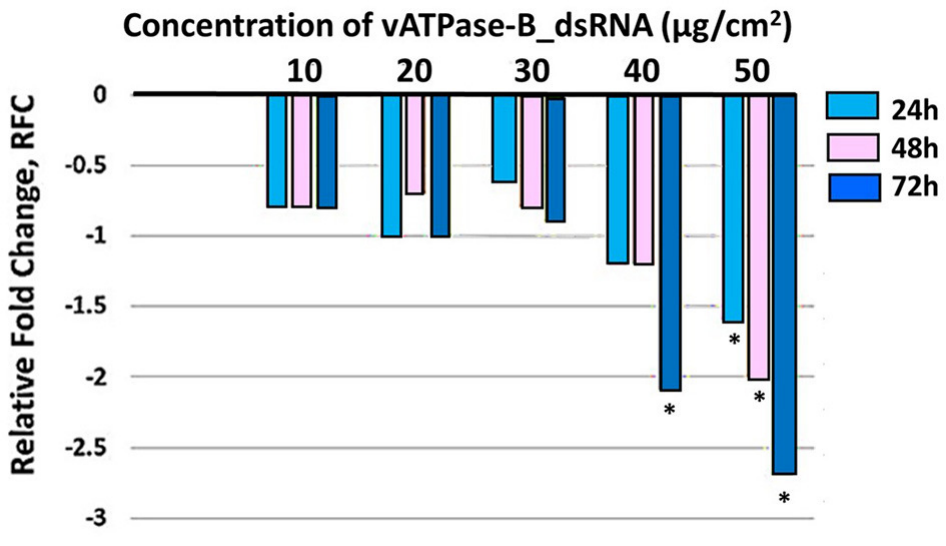
Relative fold change in expression of v-ATPase B transcript in *T. absoluta* larvae post feeding post treatment. Comparing relative expression of controls (set to “0,” to all other concentration treatments, at each of three time-points during feeding access period). RT-qPCR was used to quantify expression levels with internal control (β actin). Treatment concentrations were 10, 20, 30, 40, and 50μg/ cm^2^ leaf). Relative expression levels were determined with respect to control larvae fed on untreated tomato leaves. Analysis within each time-point across treatments compared to control was carried out by a one-way ANOVA (^*^P< 0.0001) multiple comparison test (n = 9) biological replicates, with three technical replicates.

**Figure 5:**
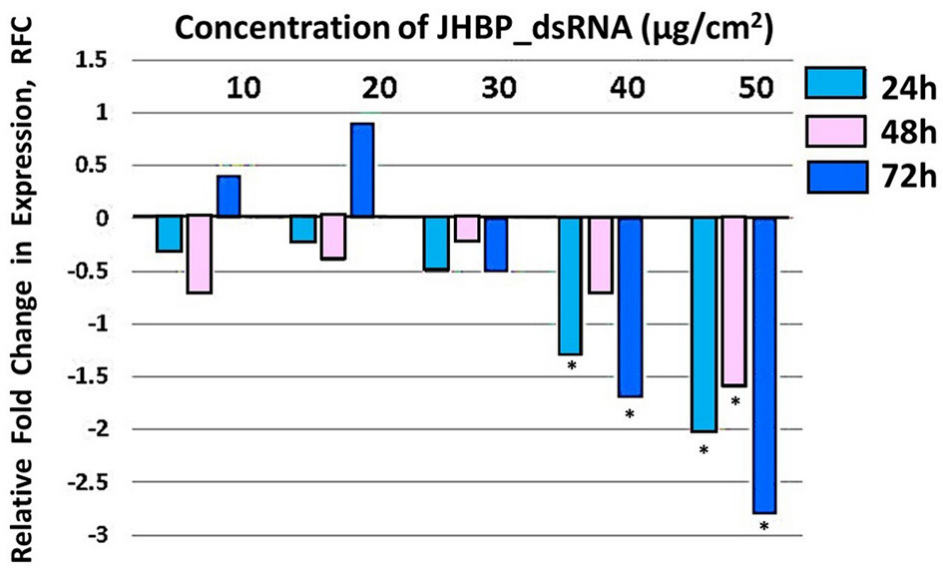
Relative fold change in expression of JHBP transcript in *T. absoluta* larvae, post feeding access on JHBP-dsRNA-treated leaf. RT-qPCR was used to access the expression levels at 24, 48, and 72 h post feeding access periods on dsRNA treatments at different concentrations (10, 20, 30, 40, and 50 μg/cm^2^ leaf). Relative expression levels were determined with respect to expression in the control at each time-point. Analysis was carried out by one-way ANOVA (^*^P < 0.0001); multiple comparison (n = 9), biological replicates with three technical replicates.

Three *T. absoluta* larvae were given feeding access periods of 24 and 48 h on single leaflets (*n* = 3), with three leaflets per treatment (*n* = 9), per concentration of dsRNA triggers (10, 20, 30, 40, and 50 μg/cm^2^), of *v-ATPase B* or *JHBP* sequences, topically applied to leaflets. Significant larval mortality was observed in larvae feeding at the increasing concentrations of each dsRNA (Figure 1, JHBP; Figure 2, v-ATPase B and LC_50_ values of 3.426 and 4.121 μg/μl; 5.126). The increased mortality rates were observed at all three feeding access periods, 24, 48, and 72 h. The treatment leaf damage decreased as the dsRNA concentration increased (Figure 3). The delivery of dsRNA triggers at different concentrations (10, 20, 30, 40, and 50μg/cm^2^) per treatment were sampled at three time-points during the feeding access period (24, 48, and 72 h). Results showed that the expression of both *v-ATPase B* and JHBP transcripts decreased progressively with increasing concentrations of dsRNA represented in Relative Fold Change per treatment (Figures 4, 5). The qRT-PCR analysis showed that relative expression of each target gene was significantly downregulated (~3.1 fold for JHBP; ~2.6 fold for vATPase B).

## Discussion

There is an urgent need to develop alternative strategies for *Tuta absoluta* pest control. In the present study, we demonstrate that oral delivery and RNAi-based silencing of JHBP and v-ATPase B transcripts cause significantly increased mortality in *T. absoluta*. Juvenile Hormone (JH) regulation is essential for development and reproductive maturation in insects (REF). In hemolymphs, JH appears complexed with a glycoprotein, the juvenile hormone-binding protein (JHBP), which serves as a carrier to release the hormone to target cells at appropriate developmental points. RNAi silencing of JHBP is reported to significantly increase mortality in several lepidopteran pests including *Helicoverpa armigera* (Lepidoptera: Noctuidae) fed on transgenic cotton. However, the JH gene family has not been extensively studied with only a few genes thus far demonstrated to be efficient targets for pest control (Yu et al., 2013).

The function of v-ATPase plays an essential role in the Lepidoptera midgut by keeping the midgut lumen alkaline and energizing secondary amino acid absorption. It is present at high density across the plasma membrane (Vitavska et al., 2003, 2005). The major challenge to implementing an effective RNAi strategy for controlling agricultural pests involves reliable delivery of dsRNA into the insects and choice of effective target genes that can confer pest protection. The use of RNAi in crop production requires delivery systems to provide dsRNA continuously as a diet component that is ingested by insects, either through transgenic plants expressing hairpin triggers, or by application of exogenous dsRNA by topical sprays, soil applications, baits, etc. (see reviews: Bramlett et al., 2020; Dubrovina et al., 2020; Fletcher et al., 2020; Kunte et al., 2020; Samada and Tambunan, 2020). Our results support previous RNAi studies on *T. absoluta* which demonstrated a high sensitivity to dsRNA triggers including Camargo et al. (2016) who first showed that RNAi of v-ATPase A could reduce *T. absoluta* in treated tomato. RNAi caused suppression and significant mortality of *T. absoluta* after feeding. And the report by Bento et al. (2020) demonstrated increased mortality of larvae *T. absoluta* fed on diets containing bacterial-expressed dsRNA to six different target genes (juvenile hormone inducible protein (JHP); juvenile hormone epoxide hydrolase protein (JHEH); ecdysteroid 25-hydroxylase (PHM); chitin synthase A (CHI); carboxylesterase (COE); and arginine kinase (AK). Finally, Rahmani and Bandani (2021) reported on the RNAi efficiency of silencing v-ATPase A in *T. absoluta* as an effective biopesticide. In the lepidopteran *Helicoverpa armigera* similar results using RNAi silencing of JHBP were reported (Ni et al., 2017).

Success of RNAi for pest control is dependent upon the choice of suitable gene target(s) (Terenius et al., 2011). Inhibition of RNAi activity in some pests is caused by excessive enzymes produced in the mouth and/ or gut of the target insects (Allen and Walker, 2012). Additionally the strength of the RNAi activity is dependent upon the concentration of dsRNA absorbed into the cells which are expressing the mRNA targeted for silencing (Terenius et al., 2011; Burand and Hunter, 2013; Scott et al., 2013). For chewing insects, exogenously applied or plant-absorbed dsRNA can be effectively introduced into the insect system (Koch et al., 2016; Faustinelli et al., 2018; Ghosh et al., 2018; Dubrovina and Kiselev, 2019; Dubrovina et al., 2019, 2020). While 130 lepidopteran genes have been screened for RNAi silencing, only 48% of these genes were silenced at a significant level, while 14% of the attempts resulted in failure (Terenius et al., 2011). Rebijith et al. (2015) reported that oral delivery of dsRNA, when effective, offers the best prospects for pest control under field conditions.

Modern agricultural biotechnologies are the most likely solution to growing demands for food, feed, and fibers, providing safer, more specific management of pathogens and pests (Adeyinka et al., 2020; Bramlett et al., 2020; Kunte et al., 2020; Raybould and Burns, 2020; Yan et al., 2020a,b; Sarmah et al., 2021). Furthermore, the increasing public acceptance and safety record (Kleter, 2020; Papadopoulou et al., 2020) continue to provide evidence for their adoption in the management of insect pests (Khalid et al., 2017; Sinisterra-Hunter and Hunter, 2018; ISAAA, 2019; Jalaluddin et al., 2019; Samada and Tambunan, 2020). Our results further support the concept of using RNAi to improve the management of *T. absoluta* (Camargo et al., 2016; Rahmani and Bandani, 2021; Sarmah et al., 2021) and other lepidopteran pests (Ni et al., 2017; Bramlett et al., 2020; Yan et al., 2020a). The capacity to use RNAi to reduce *T. absoluta* through exogenously treated leaves, plants, or diets with bacterial-expressed dsRNA provides ample evidence for moving forward toward commercialization of RNAi biopesticides for the management of this economically devastating lepidopteran pest. Furthermore, development of an exogenously applied treatment could be readily applied to many crop plants making pest suppression more effective than treating just a single crop or plant species (Dubrovina and Kiselev, 2019; Jalaluddin et al., 2019). Based on these results, RNAi strategies could be effective in targeting larvae of *T. absoluta*, with either exogenously applied, or plant-expressed double-stranded RNAs (Burand and Hunter, 2013; Scott et al., 2013; Younis et al., 2014; Zhang et al., 2015; Camargo et al., 2016; Chen et al., 2018; Jalaluddin et al., 2019; Bramlett et al., 2020; Samada and Tambunan, 2020).

## Conclusion

RNAi for specific-target gene silencing through administration of double-stranded RNA (dsRNA) has been a useful tool for developing management of insect pests (Huvenne and Smagghe, 2010; Niu et al., 2018; Adeyinka et al., 2020; Christiaens et al., 2020; Romeis and Widmer, 2020; Yan et al., 2020a,b). In this study, we produced dsRNA for v-ATPase and JHBP from *T. absoluta*. Our results show that exogenous application on tomato leaves and oral ingestion of these dsRNA triggers to v-ATPase and JHBP mRNA successfully induced RNAi silencing resulting in a significant increase in larval mortality (50 μg dsRNA /cm^2^ leaf). An additional benefit from RNAi biopesticides are their demonstrated specificity to the pest target, while not harming beneficial non-target insects that may feed on treated pests, thus protecting predators (Sarmah et al., 2021), parasitoids, and pollinators like bees (Hunter et al., 2010; Tan et al., 2016; Vogel et al., 2019). With all these breakthroughs in RNAi for pest management, our study identifies two dsRNA triggers, v-ATPase B and JHBP, that may provide suitable targets for development of RNAi-based management of *Tuta absoluta*, a devastating global lepidopteran pest.

## Data Availability Statement

The original contributions presented in the study are included in the article/Supplementary Material, further inquiries can be directed to the corresponding authors.

## Author Contributions

GR, RA, and SS-N designed the research plan, drafted, revised, and formatted the manuscript. GR, RA, and BK performed the experimental works and data compilation. GR, RA, SK, BK, NP, WH, MA, ME, AA-M, AG, KK, and PK coordinated the work and discussed the results. All authors have read and agreed to the published version of the manuscript.

## Funding

The authors are grateful to DST-SERB, Government of India for providing financial support of this research (Grant No. PDF-LS/2017/001639; dated: 06/10/2017).

## Author Disclaimer

Mention of trade names or commercial products herein is solely for the purpose of providing specific information and does not imply recommendation or endorsement, to the exclusion of other similar products or services by the U.S. Department of Agriculture. USDA is an equal opportunity provider and employer.

## Conflict of Interest

The authors declare that the research was conducted in the absence of any commercial or financial relationships that could be construed as a potential conflict of interest.

## Publisher's Note

All claims expressed in this article are solely those of the authors and do not necessarily represent those of their affiliated organizations, or those of the publisher, the editors and the reviewers. Any product that may be evaluated in this article, or claim that may be made by its manufacturer, is not guaranteed or endorsed by the publisher.
