# Supplementary material for: RNA Interference Suppression of v-ATPase B and Juvenile Hormone Binding Protein Genes Through Topically Applied dsRNA on Tomato Leaves: Developing Biopesticides to Control the South American Pinworm, Tuta absoluta (Lepidoptera: Gelechiidae)
Source: Front Physiol. 2021 Nov 18;12:742871. doi: 10.3389/fphys.2021.742871 (PMC8637209; doi:10.3389/fphys.2021.742871)
Supplement: Supplementary file 1 [file Table_1.DOC]

**RNA interference suppression of v-ATPase B and juvenile hormone binding protein genes through topically applied dsRNA on tomato leaves: Developing biopesticides to control the South American pinworm, Tuta absoluta (Lepidoptera: Gelechiidae).**

**Ramkumar, G., Asokan, R., Prasannakumar, NR., Karyiyanna, B., Karthi, S., Alwahibi, MS., Elshikh, MS., Abdel-Megeed, A., Senthil-Nathan, S., Kalaivani, K., Hunter, WB., Krutmuang, P.**

**Table S1.** Primers for target and housekeeping gene

| **No.** | **mRNA** | **Application** | **Primer** | **Length** |
| --- | --- | --- | --- | --- |
| 1 | VATPase B  *T. absoluta* | PCR | F' TGTCTCTCAACCAAGATTGACA  R' CGTTCATAGATGGTGGCTAAGT | 897 |
|  |  | qRT-PCR | F' ACAACTTCGCCATCGTGTTCG  R' CGTGATGATGCGCTCGATGGT | 120 |
|  |  | dsRNA synthesis | F' TAATACGACTCACTATAGGGCTGGGTCGATTTTCAACGGAT  R' TAATACGACTCACTATAGGGACGTTGTTTGTCGCACTGGTA | 500 |
| 2 | JHBP  *T. absoluta* | PCR | F' CTACGGTTCAGAGGACAATCAC  R' GACCATCAGGATCTGCTAGAATG | 584 |
|  |  | qRT-PCR | F'TCAAAGTGGCAGATCGGACA  R' TAGTTAGCTTTGCGTTCCTCA | 120 |
|  |  | dsRNA synthesis | F' TAATACGACTCACTATAGGGCTTAGGAATCACAGTCCGAT  R' TAATACGACTCACTATAGGGCATCAGGATCTGCAAGAATG | 500 |
| 3 | β –Actin  KU872540  Lepidoptera | PCR | ATGTGCGACGACGACGTA  TTAGAAGCACTTGCGGTGG | 1131 |
|  |  | qRT-PCR | ACCGTGTACAACTCCATCATG  ATCTCCTTCTGCATCCTGTC | 80 |

**F** -Forward, **R**- Reverse
